# The clinical picture of cachexia: a mosaic of different parameters (experience of 503 patients)

**DOI:** 10.1186/s12885-017-3116-9

**Published:** 2017-02-14

**Authors:** S. Schwarz, O. Prokopchuk, K. Esefeld, S. Gröschel, J. Bachmann, S. Lorenzen, H. Friess, M. Halle, M. E. Martignoni

**Affiliations:** 1 Department of Prevention, Rehabilitation and Sports Medicine, Klinikum rechts der Isar, Technical University, Munich, Germany; 2 Department of Surgery, Klinikum rechts der Isar, Technical University, Munich, Germany; 3 Department of Hematology and Oncology, Klinikum rechts der Isar, Technical University, Munich, Germany

**Keywords:** Cancer cachexia, Clinical parameters, Clinical picture

## Abstract

**Background:**

Despite our growing knowledge about the pathomechanisms of cancer cachexia, a whole clinical picture of the cachectic patient is still missing. Our objective was to evaluate the clinical characteristics in cancer patients with and without cachexia to get the whole picture of a cachectic patient.

**Methods:**

Cancer patients of the University Clinic “Klinikum rechts der Isar” with gastrointestinal, gynecological, hematopoietic, lung and some other tumors were offered the possibility to take part in the treatment concept including a nutrition intervention and an individual training program according to their capability. We now report on the first 503 patients at the time of inclusion in the program between March 2011 and October 2015. We described clinical characteristics such as physical activity, quality of life, clinical dates and food intake.

**Results:**

Of 503 patients with cancer, 131 patients (26.0%) were identified as cachectic, 369 (73.4%) as non-cachectic. The change in cachexia were 23% reduced capacity performance (108 Watt for non-cachectic-patients and 83 Watt for cachectic patients) and 12% reduced relative performance (1.53 Watt/kg for non-cachectic and 1.34 Watt/kg for cachectic patients) in ergometry test. 75.6% of non-cachectic and 54.3% of cachectic patients still received curative treatment.

**Conclusion:**

Cancer cachectic patients have multiple symptoms such as anemia, impaired kidney function and impaired liver function with elements of mild cholestasis, lower performance and a poorer quality of life in the EORTC questionnaire. Our study reveals biochemical and clinical specific features of cancer cachectic patients.

## Background

Ongoing cachexia represents a significant factor affecting the quality of life and prognosis of cancer patients. Cachexia is present in up to 40% in early stages of patients with gastrointestinal cancers and may be involved in up to 80% cancer deaths. However, it is still difficult to identify cachectic patients, as 40–60% of cancer patients are overweight or obese, even in advanced cancer [1].

But what do we know about clinical features of cachexia patient?

Cachectic patients usually but not always demonstrate lower body mass index (BMI), which is associated with an increased risk of tumor progression [2, 3]. At the same time, other groups report that BMI is not a prognostic factor for cancer cachexia in a cohort of patients with 17% obese, 35% overweight, 36% normal weight, and 12% underweight persons [4]. Cancer cachectic patients experience numerous complications including reduced effectiveness of chemotherapy [5, 6], reduced mobility, and reduced functionality of muscle-dependent systems, such as the respiratory and cardiovascular systems, leading to decreased quality of life and survival [7–9]. Especially in older population, cancer cachexia clinical features are key predictors of one-year mortality [10]. There is a strong correlation between decreased quality of life scores and decreased physical activity, which is strongly related to weight loss [11]. It was demonstrated that cachectic patients present lower protein, albumins, and hemoglobin levels [12].

Notably, cachexia is not an incurable situation. The important message is that weight-losing patients with unresectable pancreatic cancer can attenuate their weight loss after eight weeks of intensive nutrition intervention, and weight stabilization is associated with prolonged survival and improved quality of life [13]. However, despite our growing knowledge about the pathomechanisms of this symptom complex, a whole picture of the cachectic patient is still missing.

Some studies aim to define diagnostic criteria of cancer cachexia [14]. Usually, diagnostic tools for cachexia include loss of weight and lean body mass, fatigue, anorexia, reduced physical performance (for example, total activity or 6-min walk distance) and biochemical abnormalities of c-reactive protein (CRP), albumin, and protein.

The existing concepts for the therapy of cachexia are focusing either on nutrition or physical activity. Therefore we founded a nutrition and exercise center for cancer patients in which we are focusing on the definition of the cachectic patient and combined treatment of cancer cachexia with numerous therapy options. Our aim was to evaluate the clinical characteristics such as physical activity, quality of life, clinical dates and food intake in patients with and without cachexia to get the whole picture of a cachectic patient.

### Patients

From March 2011 cancer patients of the University Clinic, “Klinikum rechts der Isar” with gastrointestinal (GI), gynecological, hematopoietic, lung and some other tumors were offered the possibility to take part in the treatment concept including a nutrition intervention and an individual training program according to their capability. We now report on the first 503 patients at the time of inclusion in the program. All parameters like physical capability, daily calorie intake or selected lab values were documented in a prospectively designed database.

The exact definition of cachexia is a debatable issue in medical literature (reviewed in [15]). We used the definition of malnutrition proposed by ESPEN (the European Society for Clinical Nutrition and Metabolism) Consensus Statement using following criteria [16]:

Weight loss (unintentional) > 10% indefinite of time, or >5% over the last three months combined with either

- BMI <20 kg/m2 if <70 years of age, or <22 kg/m2 if > 70 years of age or
- FFMI (fat-free mass index) <15 and 17 kg/m2 in women and men, respectively.

Our definition of cachexia was also according to Fearon and co-workers [17] and is used by other researchers [18]. Here, the patients are defined as having cachexia, either when they show a weight loss of 5% during the last six months, or a weight loss of 2–5% in combination with a BMI < 20, or a weight loss of 2–5%, together with the presence of sarcopenia. Sarcopenia was defined according to a report of the European working group on sarcopenia in older people (EWGSOP) using first criterion (low muscle mass) plus either second criterion (low muscle strength) or third criterion (low muscle performance) [19, 20].

## Methods

### Laboratory parameters

Blood tests (red blood cells and white blood cells counts, platelets, hemoglobin concentrations), serum electrolytes, serum creatinine, c-reactive protein (CRP), liver function tests (aspartate aminotransferase, alanine aminotransferase, alkaline phosphatase, serum bilirubin, and cholinesterase), coagulation tests, and serum albumin levels are routinely performed upon admission to the clinic.

### Performance

Endurance capacity, maximal power output (POmax) and peak oxygen uptake (VO2peak) were measured as described [21] in a submaximal incremental exercise test on a computer-controlled bicycle ergometer. A stepwise incremental exercise protocol was applied starting at 25 or 50 watts with increments of 25 watts every three minutes until volitional exhaustion or medical reasons for exercise termination were reached. The exercise was terminated prematurely in the case of significant ECG abnormalities, severe dyspnea or excessive blood pressure increase to more than 230 mmHg systolic and/or less than 110 mmHg diastolic.

### Lung function

Spirometry provided a measurement of the forced vital capacity (FVC) and the forced expiratory volume at the end of the first second of forced expiration (FEV_1_).

### Quality of life and mental health

Health-related quality of life (HRQoL) is important parameter which can predict survival. It was assessed with the 36-Item Short Form Health Survey SF-36 survey and EORTC QLQ-C30. The EORTC QLQ-C30 is a HRQoL measure specific to cancer, whereas the SF- is a generic measure [22, 23]. The EORTC QLQ-C30 is a cancer-specific measure that can capture patients’ functional status in several domains (physical, psychological, and social), their global health status/quality of life (QoL), and symptom severity [22, 23].

### Mental health

The Hospital Anxiety and Depression Scale (HADS) was used for identifying distress. There are two subscales: depression (HADS-D) and anxiety (HADS-A). The optimal cut-off point is to be ⩾8 for the identification of suspicious cases and ⩾11 for safe cases on both subscales, with a sensitivity and specificity of 0.80 on an average [24]. With a score of ⩾13, it is possible to detect 76% of the cases among cancer patients with a specificity of 0.60, whereas 95% of the cases can be detected with a score of ⩾6 (specificity 0.21) [24].

### Nutritional risk screening (NRS)

A diet record was performed to register food intake (number of meals, calories intake/day, number and kind of additional nutrition) as described [25].

### Role of the funding source

The study was in part supported by Nutricia.

### Statistical analysis

Results are expressed as median values. Statistical analyses were performed using the SPSS (version 23, SPSS Inc., Chicago) software package. Two-sided tests and a significance level of 0.05 were used. Values were compared by Mann–Whitney *U* test for independent samples.

## Results

**Fig. 1:**
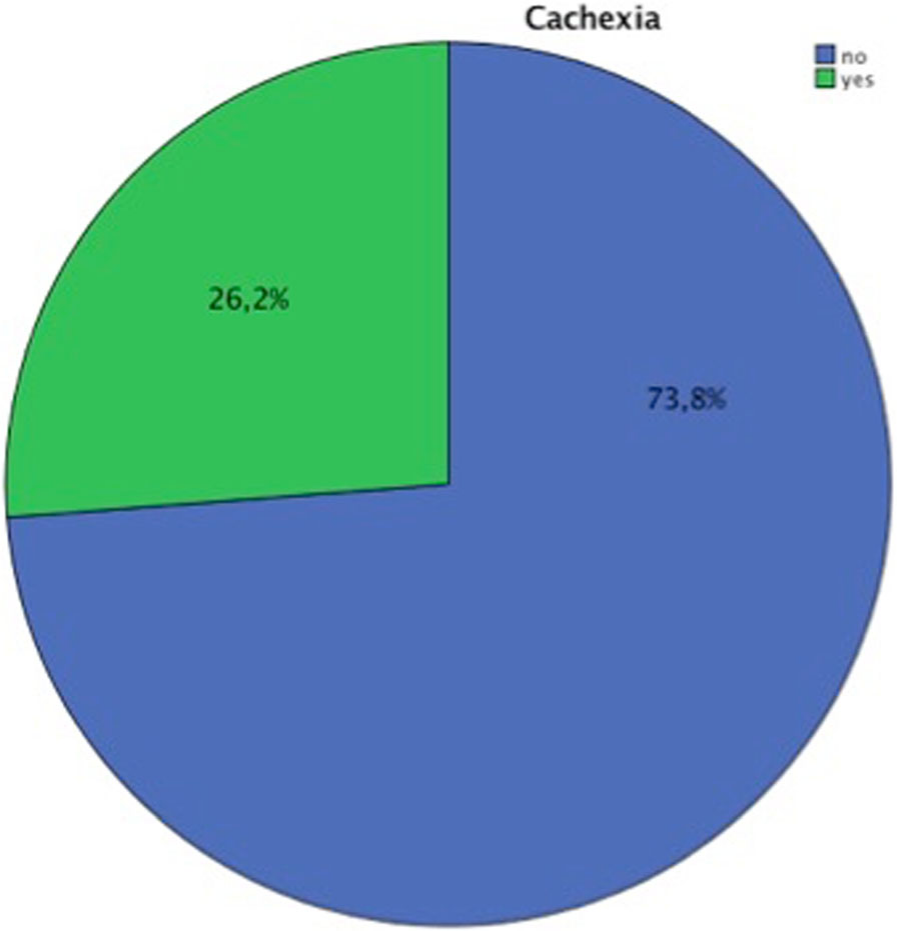
Distribution of cachectic and non-cachectic in study cohort

**Fig. 2:**
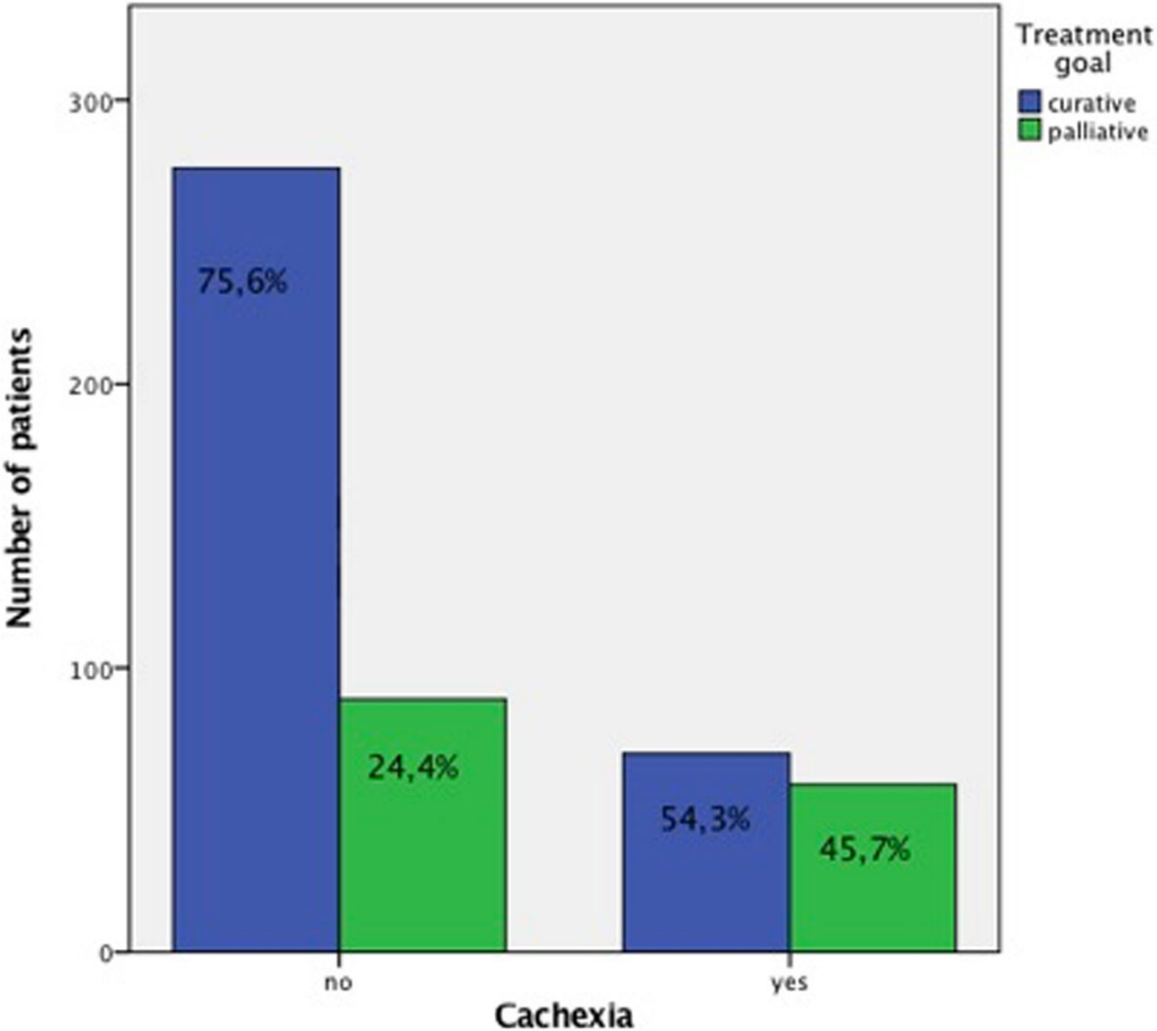
Possibility of curative treatment in cachectic patients

The parameters of the patients are noted in Table 1. One hundred thirty-one patients (26.0%) were classified as cachectic, 369 (73.4%) as non-cachectic (Fig. 1). In 3 patients (0.6%) this information was not available. As expected, cachectic patients showed pronounced weight-loss and lower values for BMI, nutrition score and Karnofsky-Index (Table 2). 54.3% of cachectic patients still receive curative treatment (Fig. 2).

**Table 1:** Characteristics of cancer patients in the analysis of cachexia

	Cachexia			
	no		yes	
	number	%	number	%
GI tumors	73	49,7%	74	50,3%
Gynecological tumors	208	89,3%	25	10,7%
Hematopoetic tumors	39	84,8%	7	15,2%
Lung tumors	11	61,1%	7	38,9%
Urological tumors	20	83,3%	4	16,7%
others	18	56,3%	14	43,8%

**Table 2:** Physical performance of the patients

	Cachexia		
	no	yes	*p*
	Median	Median	
BMI [kg/m2]	24,6	20,9	<0.001
Nutritional Risk Score	1	3	<0.001
FEV1 [l]	2,80	2,76	0.616
vital capacity [l]	3,5	3,4	0.688
IST capacity [%]	106	96	<0.001
ergometry [Watt]	108	83	<0.001
rel. performance [W/kg KG]	1,53	1,34	0.008
maximal heart frequency [/min]	153,0	145,5	0.070
Karnofsky-Index [%]	9	8	<0.001

### Laboratory variables

#### Anemia parameters

In our study hemoglobin, erythrocytes and hematocrit were significantly (*p <* 0.001) lower in cachectic patients (Table 3). Excluding patients who received chemotherapy at the time of evaluation or prior evaluation, the significant difference (*p =* 0.015) in hemoglobin level is still present (13.2 ± 1.3 g/dl for non-cachectic patients and 12.5 ± 1.5 g/dl for cachectic patients).

**Table 3:** Selected laboratory blood parameters of cancer patients in the analysis of cachexia

	Cachexia				Cachexia		
	no	yes	*p*		no	yes	*p*
	median	median			median	median	
Sodium [mmol/l]	141	140	0.007	Calcium [mmol/l]	2,39	2,33	<0.001
Kalium [mmol/l]	4,4	4,4	0,237	Albumin [g/dl]	4,50	4,30	<0.001
Creatinine [mg/dl]	,8	,8	0,042	CRP [mg/dl]	,1	,2	0.019
Urea [mg/dl]	14	15	0,217	Triglycerides [mg/dl]	113	108	0.215
AP [U/l]	70	87	<0.001	Glucose [mg/dl]	93	94	0.452
Bilirubin [mg/dl]	,4	,4	0,242	Quick [%]	100	98	0.167
GGT [U/l]	23	35	<0.001	Leukocytes [G/l]	5,39	6,03	0.054
GPT (ALAT)	25	26	0.670	Erythrocytes [T/l]	4,3	4,0	<0.001
GOT (ASAT)	27	29	0.753	Hemoglobin [g/dl]	13,1	12,0	<0.001
Cholinesterase [U/l]	7792	6703	<0.001	Hematocrit [%]	38,5	35,8	<0.001
LDH [U/l]	219	208	0.118	Thrombocytes [G/l]	232	243	0.150
Protein [g/dl]	7,0	6,8	<0.001				

#### Serum albumin und protein values

Serum albumin and serum protein were significantly decreased (*p <* 0.001) in cancer patients with cachexia (Table 3).

#### Kidney function

Both, median (0.8 mg/dl for non-cachexia and 0.8 mg/dl for cachexia, Table 3) and mean (0.85 ± 0.24 mg/dl for non-cachexia and 0.79 ± 0.19 mg/dl for cachexia) serum creatinin values were significantly lower in cachexia group (*p =* 0.042 for medians and *p =* 0.009 for means). Urinary creatinine, as well as urinary values for IgG, alpha-1-microglobulin and protein were significantly higher in cachectic patients (Table 4).

**Table 4:** Selected urinary parameters of cancer patients in the analysis of cachexia

	Cachexia		
	no	yes	*p*
	median	median	
Urinary creatinine [mg/dl]	102	169	0.001
Urinary albumin [mg/g crea]	9,9	9,9	0.053
Urinary protein [mg/g crea]	77	82	0,025
Urinary alpha-1-microglobulin [mg/g crea]	10	10	0.002
Urinary ß-NAG (U/g crea) [U/l]	5	5	0.003
Urinary IgG [mg/l]	4,3	6	<0.001

#### Liver function and parameters of protein synthesis

Two cholestasis enzymes, alkaline phosphatase (ALP) and gamma glutamyl transpeptidase (GGT), were significantly increased in cancer patients with cachexia (Table 3). The parameters of hepatocyte integrity, aspartate aminotransferase (AST) and alanine aminotransferase (ALT), were not changed. Markers of liver synthesis function cholinesterase (CHE), serum albumin and serum protein, were significantly decreased (*p <* 0.001). Totally, 187 patients (37% of all study participants) received chemotherapy at the moment of inclusion in this study, 63 (33.7%) in cachexia group (this information was not available in 2 of patients) and 124 (66.3%) patients in non-cachexia group (this information was not available in 4 patients). A significant correlation was seen between AP and current chemotherapy (*r* = 0.258, *P <* 0.001), GGT and current chemotherapy (*r* = 0.205, *P <* 0.001), as well as CHE and current chemotherapy (*r* = − 0.182, *P <* 0.001). 66 (50.4%) cachexia and 245 (66.4%) non-cachectic patients did not receive chemotherapy at the moment of inclusion in this study. In this group there is still a significant difference between cachexia and non-cachexia regarding AP (*p <* 0.001), CHE (*p <* 0.001), Quick (*p <* 0.05) and serum albumin (*p <* 0.001) but not in case of GGT (*p =* 0.154).

#### Physical performance and lung function

Three parameters of endurance capacity (absolute and relative performance) were significantly lower in cachectic patients (Table 2).

The FEV1 and VC were not significantly decreased (*p =* 0.616 and *p =* 0.688 respectively), and relative VC was significantly lower in cachectic patients (Table 2).

#### Quality of life, mental health and food intake

There are significant differences between cachectic, and non-cachectic patients regarding Global Health Score, Physical Functioning Score, Role functioning score, Social functioning score, Fatigue score, Nausea & vomiting score, Appetite loss score and Diarrhoea score (*p <* 0.001).

#### Food intake

Cachectic patients understand the problem of weight loss and take more meals per day as patients without cachexia (Table 5). Cancer patients with cachexia sometimes receive more calories compared to cancer patients without cachexia (Table 6). 12.5% of cachectic patients receive already parenteral nutrition.

**Table 5:** The number of meals in cancer patients

		Cachexia			
		no		yes	
		number	%	number	%
Calories intake/day	<500 kcal/d	1	0,3%	0	0,0%
	500 – 1000 kcal/d	6	1,8%	9	8,0%
	1000 – 1500 kcal/d	54	16,3%	21	18,8%
	1500 – 2000 kcal/d	203	61,1%	55	49,1%
	2000 – 2500 kcal/d	64	19,3%	23	20,5%
	>2500 kcal/d	4	1,2%	4	3,6%
Number of meals/day	<3	21	6,0%	7	5,7%
	3 – 5	290	82,6%	75	61,0%
	>5	34	9,7%	37	30,1%
	4	3	0,9%	0	0,0%
	5	3	0,9%	4	3,3%
Additional nutrition	no	348	96,1%	62	48,1%
	yes	13	3,6%	63	48,8%

**Table 6:** Quality of life and mental health

	Cachexia				
	no		yes		
	number	median	number	median	*P*
Global Health Score	329	58	116	50	<0.001
Physical Functioning Score	331	80	117	60	<0.001
Role functioning score	331	50	113	33	0.001
Emotional functioning score	327	58	114	58	0.739
Cognitive functioning score	338	83	116	83	0.976
Social functioning score	331	67	118	50	<0.001
Fatigue score	330	56	116	67	<0.001
Nausea & vomiting score	338	0	118	0	<0.001
Pain score	327	33	116	33	0.211
Dyspnoe score	339	33	118	33	0.908
Insomnia score	340	33	118	33	0.752
Appetite loss score	338	0	118	33	<0.001
Constipation Score	337	0	120	0	0.639
Diarrhoea score	338	0	120	0	<0.001
Financial difficulties score	337	0	116	0	0.407
Score HADS depression	336	5	118	6	0.172
Score HADS anxiety	339	7	116	6	0.104
SF36 role – physical	316	75	110	65	<0.001
SF36 physical role function	317	25	112	0	0.003
SF36 physical pain	324	62	114	57	0.572
SF36 general health	323	57	111	50	0.007
SF36 vitality	333	45	114	40	0.003
SF36 social function	332	69	118	50	0.002
SF36 role–emotional	313	67	111	67	0.397
SF36 physical well-being	327	64	111	60	0.377

**Fig. 3:**
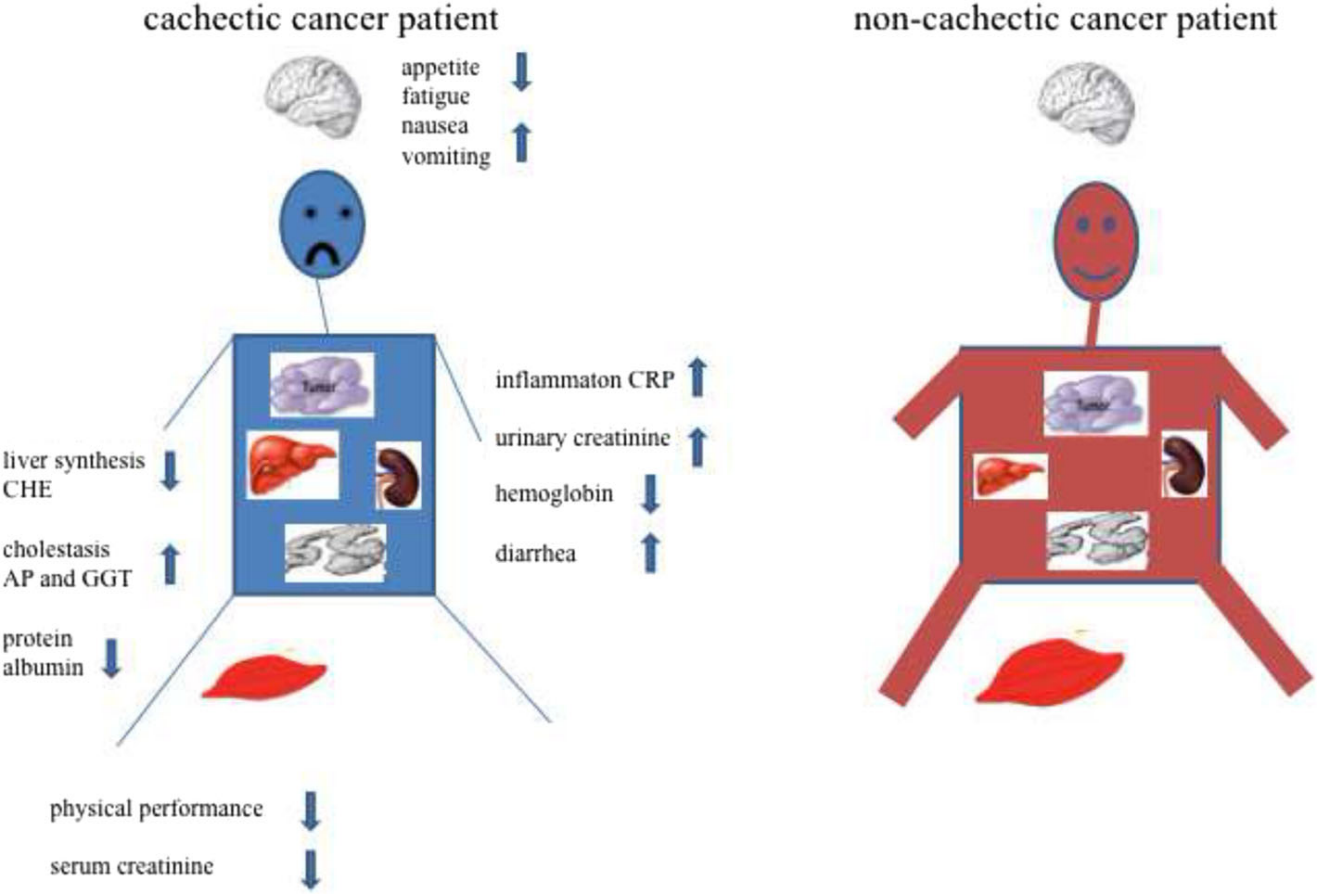
Schematic clinical picture of the cachectic patient

A summary of the clinical parameters of the cachectic cancer patient is shown in Fig. 3.

## Discussion

Our study demonstrated that cancer cachectic patients have multiple symptoms such as anemia, impaired kidney function and impaired liver function along with elements of mild cholestasis. Cachexia patients have low level of protein and albumin. As a result significantly more extracellular water and less intracellular water, compared to patients without cachexia. This means that not only low calories but also low oncotic pressure because of low protein play an important role in weight loss in cachectic patients. In parallel to protein deficiency, cachectic patients have lower performance parameters. The low levels of serum albumin, hematocrit, and fibrinogen are well-known for cachectic patients but probably not specific. Furthermore, the performance status of cachectic patients – measured by ergometry - was significantly reduced, leading to a poorer quality of life in the EORTC questionnaire (Fig. 3).

Fearon and co-workers described a population consisting of 170 advanced pancreatic cancer cachectic patients using Karnofsky Performance Score, grip strength, dietary intake, quality-of-life assessment with EuroQol EQ-5D and QLQ-C30, CRP, and CA19-9, but they were mostly concentrated on evaluation whether a 3-factor profile incorporating weight loss, low food intake, and systemic inflammation might relate better to a patient’s overall prognosis than will weight loss alone [14]. Wallengren and co-workers report on 405 patients about cachexia criteria like body mass index (BMI), weight loss, fatigue, Karnofsky performance score, physical function measured on a treadmill, low handgrip strength, lean tissue depletion (DXA or arm muscle circumference), quality of life measured by QLQ-C30 and abnormal biochemistry (inflammation, anemia, or low serum albumin) [26]. The biggest data set with 8160 patients was reported by Martin and co-workers [3], but the authors were mainly focused on BMI and % weight loss about overall survival to develop a grading system. Takayama and co-workers analyzed 406 stage IV NSCLC patients using handgrip strength, quality of life, Karnofsky Performance Scale, biochemical parameters (white blood cell count, hemoglobin, protein, albumin, triglycerides, calcium, CRP, and Insulin-like growth factor-1) and survival [27]. In the study of Theresen and co-workers 77 patients with advanced colorectal carcinoma were described using clinical parameters such as energy intake, the skeletal muscle mass cross-sectional area, a tool for assessing nutritional status the Subjective Global Assessment (SGA), protein, albumin and CRP [18].

### Laboratory variables

#### Anemia parameters

In our study population, the median hemoglobin was 12 g/dl and mean hemoglobin was 11.8 ± 1.5 g/dl. Our data regarding anemia in cachectic patients are by other groups. It was additionally reported using univariate Cox proportional hazard regression that hemoglobin was significantly associated with mortality risk [28]. According to CACHEXIA score of Argiles and co-workers [29], a tool for staging cachectic patients, hemoglobin in cachectic patients should be below 12 g/dl.

#### Serum albumin und protein values

Although we observed hypoalbuminemia and hypoproteinemia in cachectic patients, these changes were not severe. Additionally, we observed that calcium level in cachectic patients was lower than in non-cachectic patients. Taking into consideration that half of circulating calcium ions are bound to albumin, this effect resulted probably from hypoalbuminemia. Reasons for hypoalbuminemia are usually decreased synthesis, increased degradation, or an increased transcapillary escape rate [30]. We hypothesize that the primary mechanism was decreased synthesis what is supported through decreased liver synthesis function measured using liver cholinesterase (Table 3). At the same time decreased degradation was not observed because urinary albumin was unchanged (Table 4).

According to Consensus Statement of the European Society of Clinical Nutrition and Metabolism (ESPEN), visceral proteins like serum albumin concentrations that are good indicators of disease severity and outcome should not be used for either screening or diagnosis of malnutrition because of a low grade of nutrition specificity [16].

#### Kidney function

In our study there was a significant difference in serum creatinine in cachectic and non-cachectic groups that is by data of another working group [31], demonstrating that serum creatinine can be a biomarker of skeletal muscle mass in chronic kidney disease. The urinary excretion of enzymes, in particular, N-acetyl-beta-D-glucosaminidase (NAG) and alpha-1-microglobulin, non-invasive parameters of the renal tubular function, were significantly higher in cachectic patients.

#### Impaired liver function in cachexia

Two cholestasis markers, AP and GGT, were raised in cachectic patients in isolation with normal bilirubin. Though non-liver causes of this elevation like bone metastases, hyperparathyroidism, renal impairment and Paget’s disease are possible, the combination of two markers makes liver problems more likely. One possible explanation is the hepatotoxic effect of the chemotherapy confirmed by the correlation between AP, GGT, CHE and chemotherapy at the time of inclusion. The difference in AP, GGT, CHE between chemotherapy patients and chemotherapy-naive patients were not significant in our study. To our knowledge, elevated cholestasis markers and decreased liver synthesis parameters were not described in cancer cachexia until now. This elevation was mild but present in cachexia in patients under chemotherapy and without chemotherapy. Only for cardiac cachexia, it was demonstrated that 60% of cachectic patients present with abnormal cholestatic parameters [32]. Some authors proposed the importance of the role of liver enzymes in cancer cachexia (reviewed in [33, 34]) when a flow of amino acids from skeletal muscle to the liver occurs and serves for gluconeogenesis and acute-phase protein synthesis. It was suggested that an interaction between the tumor, peripheral blood mononuclear cells, and the liver may play a central role in the development and regulation of cachexia [35]. The important role of the liver in cancer cachexia was proposed by Lieffers and co-workers [36]. They hypothesized that a viscerally driven cachexia syndrome in patients with colorectal cancer originates from an increase in mass of high-metabolic-rate tissues, such as the liver and spleen.

#### Inflammation parameters (CRP) in cachexia

Increased CRP is supposed to be a valid laboratory and clinical marker in cachexia [5, 14, 37, 38]. Fearon and co-workers proposed that inclusion of a marker of systemic inflammation (e.g., CRP) in a cachexia stratification system could account for patients with real loss of function also perceiving themselves to have reduced function [14]. Though we saw a significant difference in CRP-value between cachexia and non-cachectic patients. This difference (0.1 mg/dl versus 0.2 mg/dl) is non-specific to provide additional information to the clinician when other accessible markers, such as serum hemoglobin or cholinesterase are considered. In spite of some prognostic scores for the assessment and treatment of cancer cachexia, like the Glasgow Prognostic Score (GPS) [39] or the cachexia score (CASCO) [29], which are based on CRP and albumin values, we agree with Utech and co-workers who suggest that inflammatory markers may not necessarily improve our ability to predict survival when cancer staging, serum albumin, and weight loss history are available [28]. Additionally, we think that CRP is not necessarily a characteristic parameter in cancer cachexia because it is not routinely measured in clinical practice, in Germany usually only if indicated.

#### Physical performance

Two parameters of endurance (capacity performance and relative performance) were significantly lower in cachectic patients. The dramatic change in cachexia was 23% reduced capacity performance (108 Watt for non-cachectic-patients and 83 Watt for cachectic patients) and 12% reduced relative performance (1.53 Watt/kg for non-cachectic and 1.34 Watt/kg for cachectic patients) in ergometry test.

#### Quality of life, mental health and food intake

Our results demonstrated that cachexia leads to a reduced quality of life, but the mental health is still stable. The mean value for global quality of life score was 55.7 ± 20.0 for non-cachectic patients and 47.7 ± 21.6 for cachectic patients, which is worse than the EORTC reference value global score of 61.3 ± 24.2 for all cancer types, and worse than values in other studies (for example, 68.73 ± 19.05 for patients with different cancer types on chemotherapy [40]).

These data are of special importance because for the EORTC QLQ-C30, both the general health and functioning scales and symptom scales (Dyspnea and Appetite Loss), as well as for the SF-36, role – emotional, general health, energy/vitality, and social functioning significantly predicted survival [23].

Fearon and co-workers report that weight loss alone (≥10%) did not define a population that differed from self-reported functional aspects of quality of life [14]. With our present study, we were able to demonstrate slight but significant changes in quality of life in cachectic patients without using CRP as a diagnostic parameter for cachexia. This could be explained by the different patient populations (pancreatic cancer patients that were not considered suitable to receive systemic chemotherapy in the study of Fearon and co-workers, and patients with mixed cancers in our population).

#### Food intake

It is supposed that a reduction in food intake is common in patients with progressive cancer and cachexia. Dysphagia, nausea, xerostomia and changes in taste and smell may lead to diminished food intake and thereby insufficient energy intake (reviewed in [1]). Our data show that weight loss didn’t depend on calories because cachectic patients know their problem and eat appropriately after a medical recommendation. Additionally, doctors recognize the problem of under-nutrition and prescribe parenteral nutrition (in 12.3% of patients in our cohort of cachectic patients). Tsoli and colleagues confirm our result in murine model and report that not only reduced food intake but dysregulated expression of transcription factors that control lipid metabolism and thermogenesis in brown adipose tissue lead to weight loss during the development of cachexia [41]. So, despite the same amount of meals per day, patients with cachexia had a reduced calorie intake.

#### Limitations

One potential limitation of this study was the observational design, so there may be bias inherent in who ultimately was referred to our nutrition-exercise center or decided to participate in the study. Totally, 187 (37%) patients received chemotherapy at the moment of inclusion in this study. This fact could influence the characteristics of patients. The patients are inhomogeneous regarding the type of cancer. However, future studies should be done in more homogenous cancers patient populations.

## Conclusion

Our study reveals biochemical and clinical specific features of cancer cachectic patients. The positive feature of our study is that it was conducted on large study groups (369 patients without cachexia and 131 patients with cachexia).

We were able to demonstrate that the problem of cachectic patients is not the calorie intake but protein turnover and maybe disorder in fat metabolism. Therefore we postulate that cachectic patients should be treated as high-risk patients and propose that after diagnosis of cachexia the patients should be presented to a cachexia team including “leading doctor” (for, example a surgeon, oncologist or internist, who supervises the treatment), nutritional specialist, clinical pharmacist, sports scientist and psychiatrist.

## Abbreviations

BMI: Body mass index
CASCO: Cachexia score
CRP: C-reactive protein
FEV_1_: Forced expiratory volume at the end of the first second of forced expiration
FVC: Forced vital capacity
GI: Gastrointestinal
GPS: Glasgow Prognostic Score
HRQoL: Health-related quality of life
POmax: Maximal power output
QoL: Quality of life
VO2peak: Peak oxygen uptake

## References

[1] Ryan AM, Power DG, Daly L, Cushen SJ, Ni Bhuachalla E, Prado CM. Cancer-associated malnutrition, cachexia and sarcopenia: the skeleton in the hospital closet 40 years later. Proc Nutr Soc. 2016;1–13. 10.1017/S002966511500419X 26786393

[2] Renfro LA, Loupakis F, Adams RA, Seymour MT, Heinemann V, Schmoll HJ (2016). Body Mass Index Is Prognostic in Metastatic Colorectal Cancer: Pooled Analysis of Patients From First-Line Clinical Trials in the ARCAD Database. J Clin Oncol.

[3] Martin L, Senesse P, Gioulbasanis I, Antoun S, Bozzetti F, Deans C (2015). Diagnostic criteria for the classification of cancer-associated weight loss. J Clin Oncol.

[4] Martin L, Birdsell L, Macdonald N, Reiman T, Clandinin MT, McCargar LJ (2013). Cancer cachexia in the age of obesity: skeletal muscle depletion is a powerful prognostic factor, independent of body mass index. J Clin Oncol.

[5] Punzi T, Fabris A, Morucci G, Biagioni P, Gulisano M, Ruggiero M (2012). C-reactive protein levels and vitamin d receptor polymorphisms as markers in predicting cachectic syndrome in cancer patients. Mol Diagn Ther.

[6] Dewys WD, Begg C, Lavin PT, Band PR, Bennett JM, Bertino JR (1980). Prognostic effect of weight loss prior to chemotherapy in cancer patients. Eastern cooperative oncology group. Am J Med.

[7] Caillet P, Liuu E, Raynaud Simon A, Bonnefoy M, Guerin O, Berrut G, Lesourd B, Jeandel C, Ferry M, Rolland Y, Paillaud E. Association between cachexia, chemotherapy and outcomes in older patients: A systematic review. Clin Nutr. 2016; S0261-5614(16)31344-9. 10.1016/j.clnu.2016.12.003 28017447

[8] Bachmann J, Heiligensetzer M, Krakowski-Roosen H, Buchler MW, Friess H, Martignoni ME (2008). Cachexia worsens prognosis in patients with resectable pancreatic cancer. J Gastrointest Surg.

[9] Vaughan VC, Martin P, Lewandowski PA (2013). Cancer cachexia: impact, mechanisms and emerging treatments. J Cachex Sarcopenia Muscle.

[10] Bourdel-Marchasson I, Diallo A, Bellera C, Blanc-Bisson C, Durrieu J, Germain C (2016). One-year mortality in older patients with cancer: development and external validation of an MNA-based prognostic score. PLoS One.

[11] Fouladiun M, Korner U, Gunnebo L, Sixt-Ammilon P, Bosaeus I, Lundholm K (2007). Daily physical-rest activities in relation to nutritional state, metabolism, and quality of life in cancer patients with progressive cachexia. Clin Cancer Res.

[12] Bachmann J, Friess H (2008). Martignoni ME: [Molecular mechanisms and its clinical impact in cancer cachexia]. Z Gastroenterol.

[13] Davidson W, Ash S, Capra S, Bauer J (2004). Cancer Cachexia Study G: Weight stabilisation is associated with improved survival duration and quality of life in unresectable pancreatic cancer. Clin Nutr.

[14] Fearon KC, Voss AC, Hustead DS (2006). Cancer Cachexia Study G: Definition of cancer cachexia: effect of weight loss, reduced food intake, and systemic inflammation on functional status and prognosis. Am J Clin Nutr.

[15] Mueller TC, Bachmann J, Prokopchuk O, Friess H, Martignoni ME (2015). Molecular pathways leading to loss of skeletal muscle mass in cancer cachexia - can findings from animal models be translated to humans?. BMC Cancer.

[16] Cederholm T, Bosaeus I, Barazzoni R, Bauer J, Van Gossum A, Klek S (2015). Diagnostic criteria for malnutrition - An ESPEN Consensus Statement. Clin Nutr.

[17] Fearon K, Strasser F, Anker SD, Bosaeus I, Bruera E, Fainsinger RL (2011). Definition and classification of cancer cachexia: an international consensus. Lancet Oncol.

[18] Thoresen L, Frykholm G, Lydersen S, Ulveland H, Baracos V, Prado CM (2013). Nutritional status, cachexia and survival in patients with advanced colorectal carcinoma. Different assessment criteria for nutritional status provide unequal results. Clin Nutr.

[19] Bahat G, Tufan A, Tufan F, Kilic C, Akpinar TS, Kose M, et al.: Cut-off points to identify sarcopenia according to European Working Group on Sarcopenia in Older People (EWGSOP) definition. Clinical nutrition 2016. 10.1016/j.clnu.2016.02.002 26922142

[20] Cruz-Jentoft AJ, Baeyens JP, Bauer JM, Boirie Y, Cederholm T, Landi F (2010). Sarcopenia: European consensus on definition and diagnosis: report of the European working group on sarcopenia in older people. Age Ageing.

[21] Klassen O, Schmidt ME, Scharhag-Rosenberger F, Sorkin M, Ulrich CM, Schneeweiss A (2014). Cardiorespiratory fitness in breast cancer patients undergoing adjuvant therapy. Acta Oncol.

[22] Hays RD, Morales LS (2001). The RAND-36 measure of health-related quality of life. Ann Med.

[23] Grande GE, Farquhar MC, Barclay SI, Todd CJ (2009). Quality of life measures (EORTC QLQ-C30 and SF-36) as predictors of survival in palliative colorectal and lung cancer patients. Palliative & supportive care.

[24] Singer S, Kuhnt S, Gotze H, Hauss J, Hinz A, Liebmann A (2009). Hospital anxiety and depression scale cutoff scores for cancer patients in acute care. Br J Cancer.

[25] Kondrup J, Rasmussen HH, Hamberg O, Stanga Z, Ad Hoc EWG (2003). Nutritional risk screening (NRS 2002): a new method based on an analysis of controlled clinical trials. Clin Nutr.

[26] Wallengren O, Lundholm K, Bosaeus I (2013). Diagnostic criteria of cancer cachexia: relation to quality of life, exercise capacity and survival in unselected palliative care patients. Support Care Cancer.

[27] Takayama K, Atagi S, Imamura F, Tanaka H, Minato K, Harada T, et al. Quality of life and survival survey of cancer cachexia in advanced non-small cell lung cancer patients-Japan nutrition and QOL survey in patients with advanced non-small cell lung cancer study. Supportive care in cancer: official journal of the Multinational Association of Supportive Care in Cancer 2016. 10.1007/s00520-016-3156-8 PMC4917580 27003901

[28] Utech AE, Tadros EM, Hayes TG, Garcia JM (2012). Predicting survival in cancer patients: the role of cachexia and hormonal, nutritional and inflammatory markers. J Cachex Sarcopenia Muscle.

[29] Argiles JM, Lopez-Soriano FJ, Toledo M, Betancourt A, Serpe R, Busquets S (2011). The cachexia score (CASCO): a new tool for staging cachectic cancer patients. J Cachex Sarcopenia Muscle.

[30] Fearon KC, Barber MD, Falconer JS, McMillan DC, Ross JA, Preston T (1999). Pancreatic cancer as a model: inflammatory mediators, acute-phase response, and cancer cachexia. World J Surg.

[31] Patel SS, Molnar MZ, Tayek JA, Ix JH, Noori N, Benner D (2013). Serum creatinine as a marker of muscle mass in chronic kidney disease: results of a cross-sectional study and review of literature. J Cachex Sarcopenia Muscle.

[32] Valentova M, von Haehling S, Krause C, Ebner N, Steinbeck L, Cramer L (2013). Cardiac cachexia is associated with right ventricular failure and liver dysfunction. Int J Cardiol.

[33] Argiles JM, Busquets S, Stemmler B, Lopez-Soriano FJ (2014). Cancer cachexia: understanding the molecular basis. Nat Rev Cancer.

[34] Argiles JM, Stemmler B, Lopez-Soriano FJ, Busquets S (2015). Nonmuscle tissues contribution to cancer cachexia. Mediat Inflamm.

[35] Martignoni ME, Dimitriu C, Bachmann J, Krakowski-Rosen H, Ketterer K, Kinscherf R (2009). Liver macrophages contribute to pancreatic cancer-related cachexia. Oncol Rep.

[36] Lieffers JR, Mourtzakis M, Hall KD, McCargar LJ, Prado CM, Baracos VE (2009). A viscerally driven cachexia syndrome in patients with advanced colorectal cancer: contributions of organ and tumor mass to whole-body energy demands. Am J Clin Nutr.

[37] Skorokhod A, Bachmann J, Giese N, Martignoni ME, Krakowski-Roosen H (2012). Real-imaging cDNA-AFLP transcript profiling of pancreatic cancer patients: Egr-1 as a potential Key regulator of muscle cachexia. BMC Cancer.

[38] Batista ML, Peres SB, McDonald ME, Alcantara PS, Olivan M, Otoch JP (2012). Adipose tissue inflammation and cancer cachexia: possible role of nuclear transcription factors. Cytokine.

[39] Douglas E, McMillan DC (2014). Towards a simple objective framework for the investigation and treatment of cancer cachexia: the Glasgow prognostic score. Cancer Treat Rev.

[40] Vergara N, Montoya JE, Luna HG, Amparo JR, Cristal-Luna G (2013). Quality of life and nutritional status among cancer patients on chemotherapy. Oman Med J.

[41] Tsoli M, Moore M, Burg D, Painter A, Taylor R, Lockie SH (2012). Activation of thermogenesis in brown adipose tissue and dysregulated lipid metabolism associated with cancer cachexia in mice. Cancer Res.

